# Durability of the DETOUR System for Long and Complex Femoral-Popliteal Lesions: 3-Year Results From the DETOUR2 Trial

**DOI:** 10.1016/j.jscai.2026.105457

**Published:** 2026-06-23

**Authors:** Sean P. Lyden, Peter A. Schneider, Ami Sood, Erik Zendejas, Peter A. Soukas, Arthur Tarricone, Prakash Krishnan, DETOUR2 Trial Investigators

**Affiliations:** a Department of Vascular Surgery, Heart, Vascular & Thoracic Institute, Cleveland Clinic, Cleveland, Ohio; b Division of Vascular and Endovascular Surgery, University of California, San Francisco, California; c Endologix LLC, Irvine, California; d The Miriam Hospital/Brown Medical School, Providence, Rhode Island; e Department of Orthopedics University of Texas Health Science Center, San Antonio, Texas; f Department of Medicine/Cardiology, Icahn School of Medicine at Mount Sinai, The Zena and Michael A. Wiener Cardiovascular Institute and the Marie-Josée Henry R. Kravis Cardiovascular Health Center, Icahn School of Medicine at the Mount Sinai Hospital, New York, New York

**Keywords:** DETOUR, endovascular therapy, femoropopliteal disease, lower extremity bypass, percutaneous transmural arterial bypass, peripheral arterial disease

## Abstract

**Background:**

The DETOUR2 Trial assessed the safety and effectiveness of the DETOUR System for percutaneous transmural arterial bypass (PTAB) of the femoral-popliteal (FP) artery. Previously published data reported that the procedure met key safety and effectiveness criteria with durable results through 12 months of follow-up, demonstrating a high technical success rate, few major complications, and maintained patency at rates comparable to surgical bypass. This final report describes the 36-month outcomes.

**Methods:**

The DETOUR2 Trial evaluated a novel PTAB technique for treating long-segment (>200 mm) FP lesions in patients with severe peripheral arterial disease (Rutherford Class 3-5) using the DETOUR System (NCT03119233). This international, multicenter, prospective, single-arm trial presents data from participants followed for 36 months.

**Results:**

Thirty-two sites enrolled 202 patients, and 200 patients were treated with the DETOUR System. Mean target lesion length was 327.1 ± 61.4 mm. Primary patency through 36 months was 58.2%. Freedom from clinically driven target lesion revascularization through 36 months was 66.8%, and freedom from major adverse limb events was 64.5%. Occurrence of deep venous thrombosis at 36 months was 4.1%, with no pulmonary emboli reported.

**Conclusions:**

PTAB with the DETOUR System provides durable results with favorable safety through three years for very long FP lesions exceeding 200 mm in length. DETOUR System patency is comparable to prosthetic FP bypass but with lower complication rates and outperforms traditional endovascular therapy in these long, complex lesions.

## Introduction

Long-segment femoropopliteal (FP) lesions are a common presentation in patients with symptomatic peripheral arterial disease. FP lesions over 200 mm in length present significant clinical challenges when treated using endovascular techniques, as they are challenging to cross, subject to frequent restenosis, and patients may be subject to multiple revascularization procedures to maintain patency. Based on guideline recommendations, the treatment of choice for TASC D lesions has been open surgical bypass.^1^, ^2^, ^3^ However, lower extremity bypass (LEB) has several limitations including availability of autologous venous conduit, increased postoperative morbidity and mortality, longer length of stay, and a lengthy recovery period as compared to endovascular approaches.^4^, ^5^, ^6^ Because many PAD patients have significant comorbidities and are at an increased risk of complications after open surgery; endovascular therapy (EVT) has become an attractive treatment paradigm for lesions over 200 mm. Limited evidence supports long-term durability of EVT in these longer FP lesions.^1^^,^^7^, ^8^, ^9^ For example, a recent systematic review of EVT studies with TASC D lesions demonstrated 3-year primary patency ranging from 12% to 51%.^9^

Percutaneous transmural arterial bypass (PTAB) is an endovascular alternative for the treatment of long-segment, complex femoropopliteal disease. The DETOUR System (Endologix LLC) includes the ENDOCROSS crossing device and TORUS Stent Grafts to create an endovascular femoropopliteal bypass (Central Illustration). PTAB with the DETOUR System uses a unique approach to exclude the long lesion and deploy covered stent grafts from the above-the-knee popliteal artery through the femoral vein, and back into the proximal SFA.^10^ This percutaneous approach reduces the risk of wound and other complications associated with open bypass surgery.

**Central Illustration:**
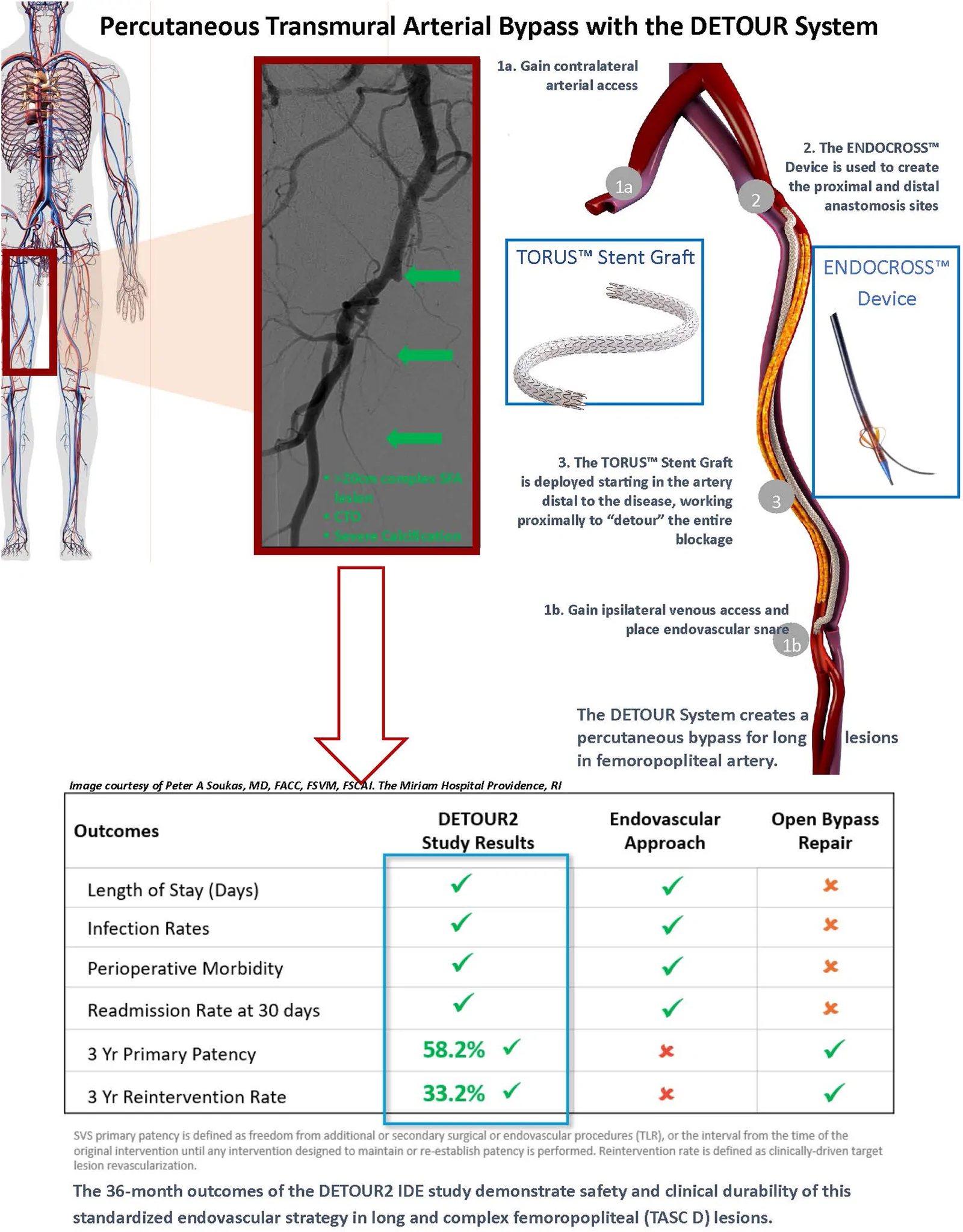
**Percutaneous transmural arterial bypass with the DETOUR System.**

The DETOUR Endovascular Technique for long OcclUsive femoropopliteal Revascularization (DETOUR2) clinical trial is a prospective investigational device study for patients with long-segment, complex FP disease. Patient demographic characteristics and results through 12 months of follow-up were previously published,^11^ and we present the final 3-year results of the DETOUR2 Study.

## Materials and methods

### Study design and administration

DETOUR2 is a prospective, multicenter, single-arm clinical investigation evaluating the safety and effectiveness of the DETOUR System for percutaneous bypass of long-segment FP (TASC D) lesions. Written consent was obtained from all patients, and the study was approved and executed in accordance with the applicable Institutional Review Board or the local Competent Authority/Ethics Committee (NCT03119233).^12^ There were 36 study sites in the United States and Europe. An independent clinical events committee and a data safety monitoring board reviewed adverse events and monitored safety (Syntactx). An independent core laboratory (Cleveland Clinic Peripheral Core Laboratory) reviewed all images. The trial description and details were previously published.^11^ A brief overview is presented here.

### Patient eligibility

Eligible patients had symptomatic leg ischemia, with a Rutherford-Becker Clinical Category class (RCC) 3 to 5, a Venous Clinical Severity Score (VCSS) <3 and angiographic evidence of a lesion >200 mm in length within the SFA and/or popliteal artery. At least one patent infrapopliteal vessel runoff to the foot was necessary. A comprehensive list of inclusion and exclusion criteria is described in Supplemental Table S1. The study design allowed an option to designate the initial 2 patients (prespecified) enrolled and treated at each site as roll-in subjects.

### Enrollment and procedure

To reduce selection bias, patients were evaluated for study eligibility by a screening committee based on inclusion/exclusion criteria, clinical assessment, and imaging. Final eligibility was confirmed during procedural imaging and sheath placement. The PTAB procedure followed confirmation of eligibility. Reasons for screen failure included anatomy, as well as medical reasons.

The key steps of the procedure are as follows: (1) obtain ipsilateral venous access and place an introducer sheath in a below-the-knee vein, (2) gain contralateral common femoral arterial (CFA) access, place an 8F sheath, and advance a 0.014-inch wire into the proximal SFA, (3) advance a snare into the femoral vein (FV) to the level just above the arterial lesion and 30 to 40 mm distal to the SFA/profunda bifurcation, (4) carefully position ENDOCROSS device and deploy the needle into the adjacent FV, (5) advance guidewire from artery to vein and capture with a snare, (6) externalize the wire through venous access site, (7) dilate proximal anastomosis, (8) advance ENDOCROSS device into the popliteal vein distal to the lesion in the popliteal artery, (9) the distal anastomosis is similarly created from the FV into the popliteal artery followed by wire advancement into the tibial artery, (10) once the distal anastomosis has been dilated, the TORUS stent grafts are deployed distal to proximal until the graft is flush to the profunda origin, (11) ensure adequate overlap (≥5 cm) between stent grafts, and (12) dilate the grafts and anastomoses appropriately.

### Follow-up requirements

Follow-up visits included office visits with duplex ultrasonography (DUS), functional testing, and adverse event assessments occurring at 30 days, 6-, 12-, 24-, and 36 months postprocedure. Patients were required to be on dual antiplatelet (DAPT) therapy (including aspirin ≥75 mg and clopidogrel 75 mg daily) for a minimum of 3 years, except in cases where oral anticoagulants were prescribed. Oral anticoagulation postprocedurally was optional and administered at the discretion of the physician.

### Study end points

The primary safety end point was freedom from a major adverse event (MAE) through 30 days. MAE was defined as death, clinically driven target lesion revascularization (CD-TLR), major amputation of the treated limb, symptomatic deep vein thrombosis (DVT), pulmonary embolism (PE), or procedure-related bleeding requiring any transfusion or surgery.

The primary effectiveness end point was primary patency through 12 months, defined as the absence of CD-TLR and absence of recurrent target lesion diameter stenosis >50% by imaging (eg, DUS peak systolic velocity ratio of >2.5 or invasive angiography) within the stent or 1 cm above or below the treated segment.

The study also examined several secondary safety outcomes. These included major adverse limb events (MALE), major target limb amputation, and symptomatic DVT. Changes in the VCCS Score and the Villalta scale were also monitored. Additional measures were CD-TLR, primary-assisted patency, secondary patency, and clinical improvement, which was defined as an improvement of at least one category on the RCC scale.

### Statistical analysis

Statistical analyses were described in detail previously.^11^

The intention-to-treat (ITT) cohort included all participants that were considered enrolled and underwent the PTAB procedure (excluding roll-in). The modified ITT (mITT) cohort was a subset group where a TORUS stent graft was implanted using the ENDOCROSS device. Continuous variables are described as mean and SD. Categorical variables are described as frequencies and percentages. A post hoc evaluation was conducted to provide additional insight into potential risk factors and the weight of their influence. Logistic regression models were evaluated for major outcomes. Parsimonious models were constructed and interaction terms investigated for potential synergies. The Kaplan-Meier method was used to determine rates for the 36-month data, with observations censored at the last-evaluable data point. All analyses were performed with SAS version 9.4 (SAS Institute).

## Results

### Patient demographic characteristics

Patient demographic characteristics and lesion characteristics are outlined in Table 1. The study population consisted of 220 participants, with 18 participants allocated to the initial roll-in phase and the remaining 202 forming the ITT group. Two patients in the ITT group underwent the procedure, had the ENDOCROSS device introduced into the vasculature, but did not have a TORUS stent graft implanted. These patients were excluded from the mITT group. Mean age was 68.9 ± 9.4 years, and 26.2% of patients were female. Most of the patients included in this study (59.9%) had a previous peripheral intervention, and 16.8% had a previous surgery on the target limb. Participants had various comorbidities including coronary artery disease (46%), hypertension (87.6%), diabetes (34.7%), history of smoking (91.1%), and renal insufficiency (10.9%). Mean target lesion length was 327.1 ± 61.4 mm, and 70.4% of lesions were severely calcified. Almost all lesions were considered CTO (96.0%), and 17.3% were ISR lesions. Procedural characteristics were previously described.^11^

**Table 1:** Patient demographic characteristics and lesion characteristics

Characteristics	N = 202
Age, y	68.9 ± 9
Male sex	73.8% (149/202)
Medical history/comorbidities	
Renal insufficiency	10.9% (22/202)
Hypertension	87.6% (177/202)
Diabetes mellitus	34.7% (70/202)
Hyperlipidemia	69.3% (140/202)
Coronary artery disease	46.0% (92/200)
History of smoking	91.1% (184/202)
Previous peripheral intervention	59.9% (121/202)
Previous history of surgical intervention	16.8% (34/202)
Rutherford category	
3	77.7% (157/202)
4	17.8% (36/202)
5	4.5% (9/202)
Target limb ankle-brachial index	0.61 ± 0.22 (193)
Lesion length, mm	327.1 ± 61.3
Lesion characteristics^a^	
Chronic total occlusion	96.0% (194/202)
In-stent restenosis	17.3% (35/202)
Calcification	
None/mild	29.1% (52/179)
Moderate	0.6% (1/179)
Severe	70.4% (126/179)

Values are mean ± SD or % (n/N).

a Core lab adjudicated.

### Patient follow-up compliance

Among the mITT cohort of 202 subjects, there were 17 (8.5%) deaths, 16 (8%) subjects were withdrawn, and 5 (2.5%) were lost to follow-up. DUS imaging compliance through the study ranged from 94.8% (at 3 years) through 98.9% (at 12 months).

### Safety end points

The MAE rate at 36 months was 42.3%. MAE observed in the study included 16 deaths (8.9% through 36 months), none of which were determined to be related to the procedure or device used. There were 3 cardiovascular-related deaths. Most of the deaths were categorized as noncardiovascular: 13 in total (4 general, 3 respiratory, 3 neoplasm, 1 nervous system, 1 renal/urinary, and 1 metabolism disorders). Freedom from MALE at 36 months postprocedure was 64.5%. Most of these patients underwent some type of endovascular reintervention (Table 2). When stratified by Rutherford score, freedom from MALE at 36 months was similar across classes at 64.6%, 64.0%, and 64.8% for RCC 3, 4, and 5, respectively. While acute limb ischemia (ALI) was included as part of MALE, it was not assessed as a separate end point. However, a post hoc analysis revealed 18 ALI events in 16 patients. One patient had thrombosis located proximally to, not within, the stent grafts. At least 4 ALI events happened after noncompliance/cessation of DAPT. All patients were treated successfully with various combinations of catheter-based therapies. There were no residual clinical sequelae resulting from ALI or the intervention in these patients.

**Table 2:** Additional revascularization information

Revascularization type (CD-TLR)	Follow-up period			
	Perioperative (≤30 d)	Year 1 (day 31-365)	Year 2 (day 366-730)	Year 3 (day 731-1095)
Surgery	1 (1)	5 (8)	6 (6)	2 (2)
Open bypass	-	3 (3)	5 (5)	2 (2)
Thrombectomy	1 (1)	-	1 (1)	-
Amputation	-	3 (3)	-	-
Patch angioplasty	-	2 (2)	-	-
Endovascular	1 (1)	14 (17)	12 (16)	16 (21)
Angioplasty (PTA, POBA, DCB)	-	12 (12)	12 (13)	16 (17)
Stent graft	1 (1)	5 (5)	3 (3)	3 (4)
Thrombectomy, EKOS, thrombolysis	2 (2)	13 (19)	10 (14)	10 (12)
Other or unknown	-	4 (4)	7 (8)	7 (7)

Values are number of patients (number of interventions).

CD-TLR, clinically driven target lesion revascularization; DCB, drug-coated balloon; EKOS, EkoSonic endovascular system; POBA, plain old balloon angioplasty; PTA, percutaneous transluminal angioplasty.

Over 3 years, there were 36 patients who presented with graft occlusion (Supplemental Table S2). Most (21 patients) occurred after 1 year. There were at least 8 patients that were found to have an occlusion from scheduled follow-up imaging with no symptoms present. One patient presenting with occlusion (but no ALI) resulted in ischemic rest pain and had a planned open bypass repair to address this. Only a small number (n = 5 patients) with occlusion also presented with ALI; all were resolved with catheter-based therapies with no residual clinical sequelae.

Freedom from major amputation was 97.9% at 36 months and was unchanged from 12 months. There were 3 major amputations of the treated limb. Of these, 2 of the subjects were RCC 3, and 1 subject was RCC 5 at baseline. One major amputation was due to osteomyelitis. The other 2 major amputations occurred on day 141 and day 352 due to worsening limb ischemia. One patient had a partial fifth right metatarsal amputation for osteomyelitis on day 41. This was not considered a major amputation per protocol, nor device or procedure-related, and thus not included as a MAE. The freedom from amputation rate in patients with limb threat (R4, R5) at baseline was 97.7% through 3 years.

### Effectiveness end points

**Figure 1:**
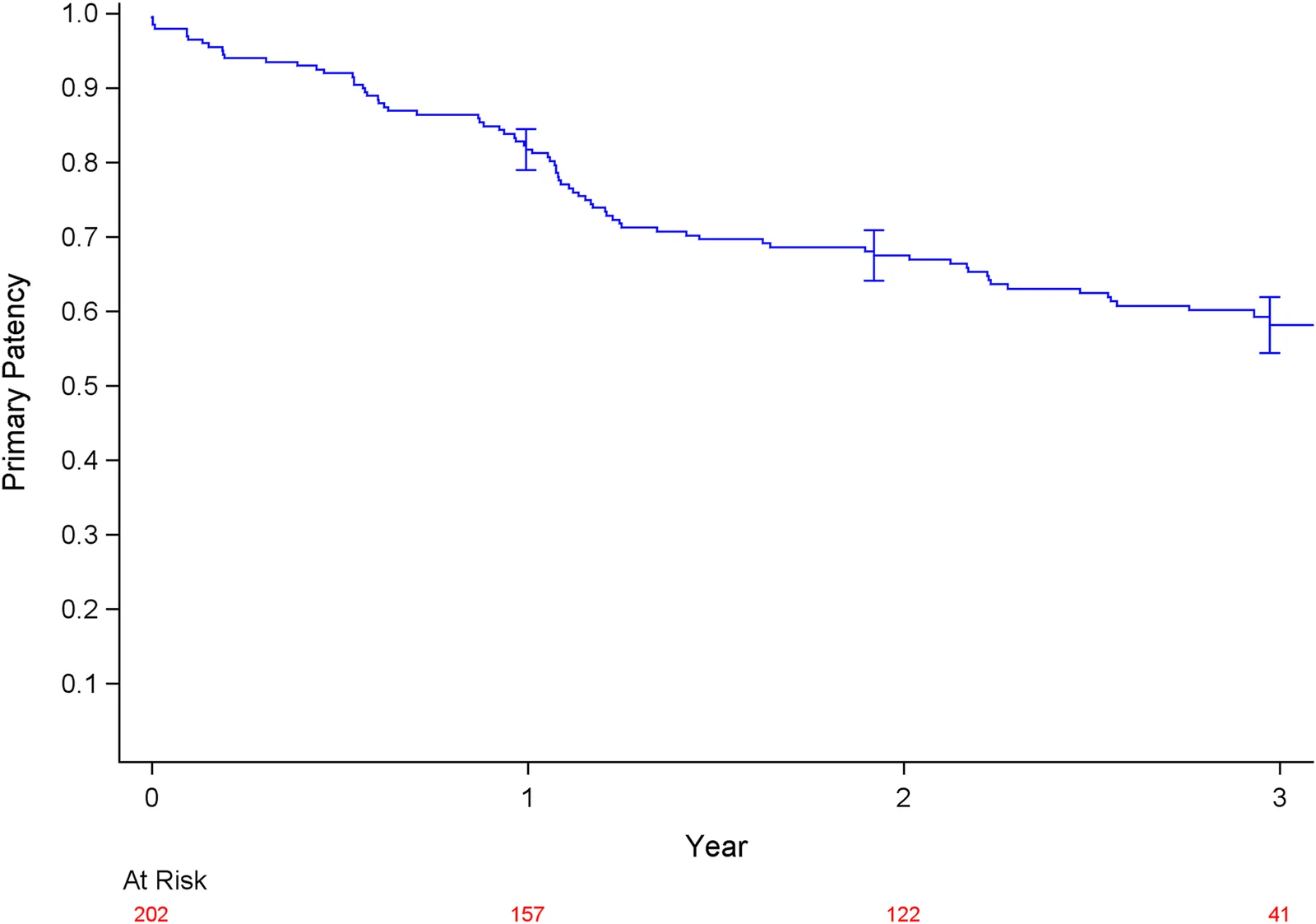
**Primary patency (mITT) through 36 months.** Primary patency 36 months was 58.2%.

**Figure 2:**
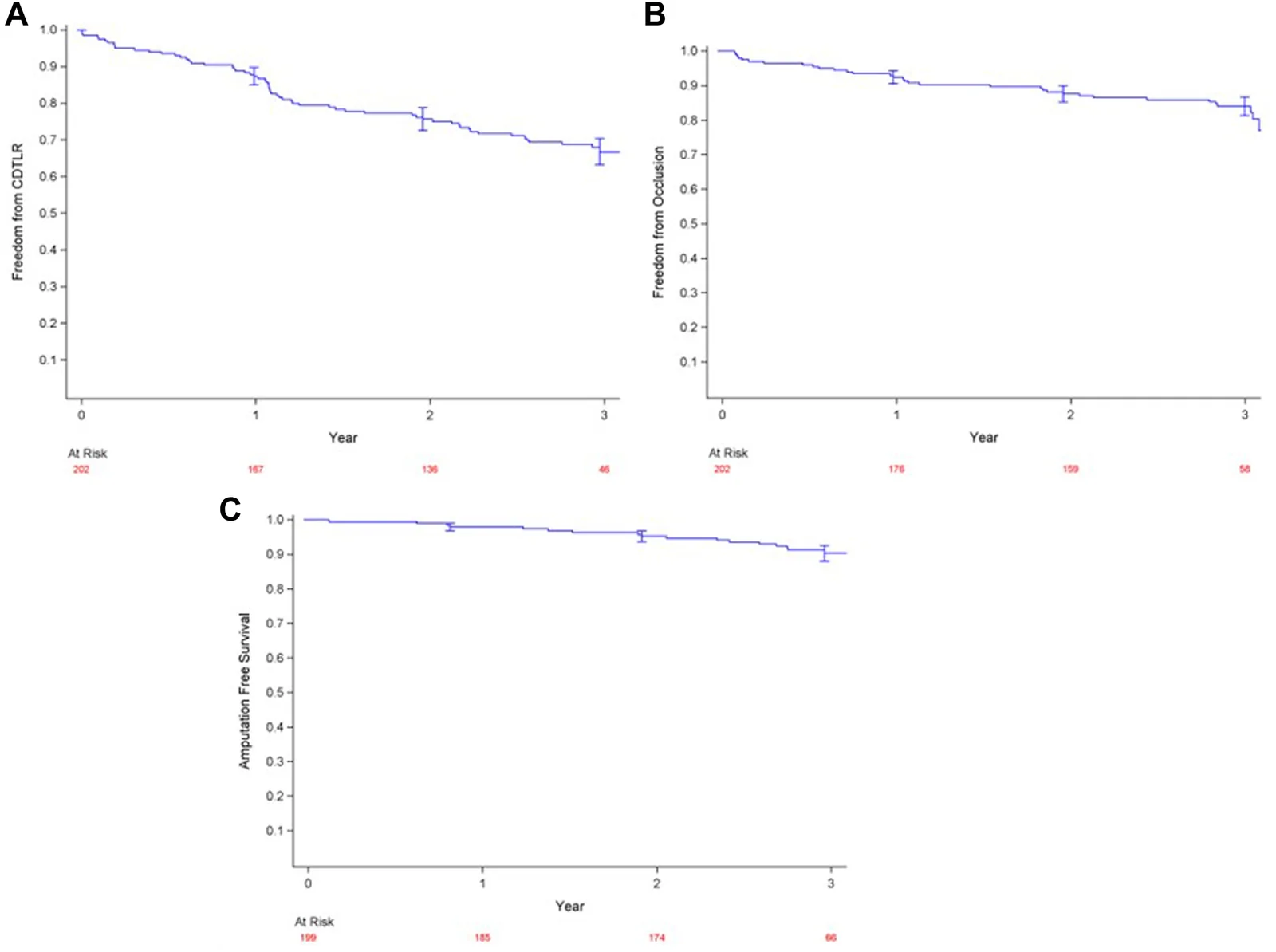
**Secondary end points through 36 months.** Kaplan-Meier estimates of (**A**) freedom from CD-TLR; (**B**) freedom from occlusion; and (**C**) amputation-free survival.

Clinical success, as assessed by improvement in RCC scale by ≥1 at 36 months, was 96.7%. Primary patency, as per SVS guidelines, through 36 months was 58.2% (Figure 1). Freedom from CD-TLR (Kaplan-Meier estimate) was 87.3% through 12 months, 75.6% through 24 months, and 66.8% through 36 months (Figure 2). Patency defined as freedom from occlusion (flow/no flow) was 92.4% at 12 months, 87.6% at 24 months, and 84.0% at 36 months (Figure 2). Amputation-free survival through 36 months was 90.3%. The change in mean ABI from baseline remained positive at 0.35 ± 0.30 during this duration.

### Venous status

The occurrence of symptomatic DVT in the affected limbs was 4.1% through 12 months and remained unchanged at 36 months without additional cases. PE also remained unchanged with no reports of an occurrence throughout the 36 months. Villalta overall score was 1.7 ± 3.12 (median: 0, IQR: 0, 2) at 36 months with a change of −0.5 ± 3.48 (median: 0, IQR: −2 ,0) from baseline. At 24 and 36 months postprocedure, the approximate overall change in VCSS from baseline was 0.3 and 0.6, respectively. At 36 months, the overall VCSS was 1.6 ± 2.55 with a change of 0.6 ± 2.45.

### Dual antiplatelet therapy compliance

At the first follow-up visit, the majority were on DAPT (79%), and 46.5% were on dual pathway inhibition (oral anticoagulant + antiplatelet therapy). Patients generally remained on the therapy they started, with 85.0% continuing aspirin, 81.6% continuing antiplatelet medications, and 28.1% on oral anticoagulants at 36 months.

### Predictors of efficacy failure

A univariate model was performed to assess predictors of efficacy failure. Baseline demographic characteristics, medical history, vascular/procedural characteristics, and medications were evaluated as potential risk factors for CD-TLR. Follow-up arterial lumen diameter measurements, including distal CFA, proximal SFA, proximal profunda, and distal SFA/popliteal arteries, were included. (Table 2). A multivariable model was then performed to adjust for confounding variables. In the final model, small distal CFA diameter of ≤6.7mm and high baseline VCSS pain scores (≥ 2) were found to be significant predictors of CD-TLR (Table 3). Each millimeter increase in distal CFA diameter above 6.7 mm led to an approximate 30% reduction in CD-TLR. Older age, smoking, and sex were found not to be significant predictors of CD-TLR.

**Table 3:** Predictors of CD-TLR

Covariate/parameter	Hazard ratio (95% CI)	*P* value
Univariate predictors		
Distal CFA diameter (mm)	0.70 (0.57-0.86)	<.001
Baseline pain score (0-3)	1.65 (1.11-2.47)	.01
Multivariable predictors		
Distal CFA diameter (mm)	0.72 (0.59-0.89)	.002
Baseline pain score (0-3)	1.52 (1.00-2.30)	.048

Smoking was significant in a multivariable approach only when the model covariables were limited to distal CFA diameter.

## Discussion

This is the first reported device study of endovascular treatment of FP lesions with a mean lesion length over 30 cm with follow-up through 36 months.^9^^,^^13^ Key findings of the DETOUR2 final results demonstrate durable efficacy in the endovascular management of TASC D FP arterial lesions through 36 months with 58.2% primary patency and freedom from CD-TLR at 66.8%. While there is moderate loss of primary patency through follow-up, similar types of studies with long lesions (with both EVT and LEB-synthetic graft) have also demonstrated similar decreases in patency.^14^, ^15^, ^16^, ^17^, ^18^ Notably, Viabahn studies have comparable patency decreases from follow-up years 1 to 3. Bohme et al found a patency decrease of 68.2% at 1 year to 40.6% at 3 years (with a mean lesion length of 26.5 cm),^16^ and Iida et al^17^ demonstrated a similar decrease from 85.3% to 69.8% (with a mean lesion length of 23.6 cm).

This study also highlighted the favorable safety with low major amputation and DVT rates which have remained unchanged since 12 months postprocedure.^11^ While these outcomes support the durability of the DETOUR System, the clinical improvements such as RCC score and ABI underscore the impact on patients and relief from their symptoms. This can correlate to improvements in walking ability, pain relief, and a risk reduction of amputation.^19^^,^^20^

Historically, societal guidelines have recommended traditional LEB as the preferred therapy for TASC D lesions.^3^ Autologous above-the-knee vein bypass remains the preferred and superior conduit for surgical revascularization; however, approximately 20% of patients lack a suitable vein, necessitating the use of prosthetic grafts.^21^ Long-term outcomes of synthetic grafts have demonstrated durability comparable to EVT, with reported patency from 46% to 57%.^14^^,^^15^^,^^18^ Thus, contemporary clinical practice guidelines increasingly endorse EVT as a “first line” revascularization option for patients irrespective of lesion length,^22^^,^^23^ reflecting its favorable clinical profile relative to LEB.

Comparative studies have highlighted the limitations of surgical bypass. A single-center study comparing open bypass with percutaneous transluminal angioplasty ± stent in patients with claudication demonstrated that surgical bypass had improved freedom from restenosis and symptom relief despite treatment of more extensive disease; however, these benefits were offset by longer hospital stays and higher rates of wound infection.^24^ The REVIVE meta-analysis of randomized controlled trials comparing EVT to surgical bypass for FP disease found no significant differences in major adverse limb events, major amputation, mortality, or amputation-free survival, while demonstrating significantly lower rates of bleeding and infection with EVT.^25^ A separate contemporary meta-analysis, focusing on chronic limb-threatening ischemia patients reported no statistically significant differences in overall patency between EVT and bypass.^26^

Longer-term studies of various EVT therapies in long lesions such as IN.PACT Global study (150 mm cohort), STELLA-SUPERA, and VIASTAR trials have shown sustained positive results.^27^, ^28^, ^29^ However, these trials evaluated substantially shorter lesions than the mean lesion length of 327 mm observed in the DETOUR2 Trial. A recent systematic review and meta-analysis of EVT studies for TASC D femoropopliteal lesions demonstrated 1-, 2-, and 3-year pooled primary patency of 67%, 48%, and 42%, respectively.^9^ In contrast, the DETOUR2 Trial demonstrated superior durability with primary patency rates of 81.8% at 12 months, 67.5% at 24 months, and 58.2% at 36 months. Notably, the 36-month DETOUR2 patency exceeded that reported in a meta-analysis of published synthetic above-the-knee prosthetic LEB patency which ranged from 21.9% to 39.3%.^30^ The durability and clinical benefits suggest that PTAB with the DETOUR System is an alternative for long lesions which offers the endovascular advantages of shorter length of stay, lower morbidity and mortality, and readmission rates with long-term durability.

There is a potential risk (real or perceived) that the DETOUR System could result in venous flow disruption, resulting in deep vein thrombosis, phlebitis, or leg swelling. The DETOUR2 Trial demonstrated rates of 2.5% and 4.1% for symptomatic DVT at 30 days and 12 months, respectively; and the PE rate was 0% through 12 months.^11^ There were no additional new symptomatic DVT or PE through 36 months. Perioperative DVT rates after LEB have been shown to be between 0.5% and 3.6%.^31^, ^32^, ^33^, ^34^ A recent Medicare claims study (including LEB and EVT with n = 31,593) demonstrated DVT and PE rates of 3.8% and 0.9% at 30 days and 4.8% and 1.2% at 90 days.^35^ DETOUR2 Trial DVT and PE rates were comparable to these published outcomes.

Another concern with the DETOUR System is the potential risk of stent thrombosis and its impact on limb prognosis. In previous EVT and LEB studies, thrombosis has been observed in 2% to 36% of patients within 6 months of stent implantation for the FP segment^36^, ^37^, ^38^, ^39^, ^40^, ^41^ and has been shown to be associated with an increased risk of MALE.^36^ While DETOUR2 did not evaluate graft thrombosis exclusively, there were 16 patients with ALI. All patients were treated successfully with catheter-based therapies with resolution and no residual clinical sequelae or worsening ischemia.

The DETOUR System was designed specifically for peripheral arterial disease with lesions that are challenging to treat, including patients who have complex lesions of the FP artery, those with previously failed endovascular procedures, and those who may not be good candidates for surgical bypass. Specifically, these may be patients with multiple comorbidities, no available vein for surgical bypass, a hostile groin, or uncrossable ISR or CTO.

As with most single-arm studies, direct comparisons of the DETOUR2 study outcomes with alternative therapies are not possible. There is also difficulty in generalizing these results to larger populations and challenges with using historical controls. Inherent limitations that may reflect bias or other compounding factors rather than treatment effects may also be present. The DETOUR2 study utilized prespecified objective performance criteria, independent imaging core labs, and safety committees to mitigate some of these limitations. Potential DETOUR2 subjects were reviewed by a screening committee to confirm eligibility to reduce patient selection bias. Although DETOUR2 did not collect functional measurement data beyond 12 months and the durability of functional benefits in DETOUR2 subjects is unknown, clinical success—considered a surrogate for pain and functional status—was 96.7% at 36 months.

Future real-world data with the DETOUR System may validate efficacy parameters and compare outcomes with contemporary practice.

## Conclusion

The trial has demonstrated durable results through 3 years with favorable patency rates surpassing EVT outcomes and comparable to prosthetic above-the-knee LEB but with low complication rates for patients with long FP lesions. The long-term safety and efficacy results of PTAB with the DETOUR System bring forward a compelling minimally invasive alternative to EVT and LEB in appropriate patients.

## CRediT authorship contribution statement

**Sean P. Lyden:** Conceptualization, Investigation, Methodology, Supervision, Writing – original draft, Writing – review & editing. **Peter A. Schneider:** Investigation, Methodology, Supervision, Writing – original draft, Writing – review & editing. **Ami Sood:** Data curation, Formal analysis, Project administration, Writing – original draft, Writing – review & editing. **Erik Zendejas:** Data curation, Formal analysis, Methodology, Project administration, Software, Validation, Writing – original draft, Writing – review & editing. **Peter A. Soukas:** Investigation, Supervision, Writing – original draft, Writing – review & editing. **Arthur Tarricone:** Formal analysis, Software, Writing – original draft, Writing – review & editing. **Prakash Krishnan:** Formal analysis, Supervision, Writing – original draft, Writing – review & editing.
