# Supplementary material for: Durability of the DETOUR System for Long and Complex Femoral-Popliteal Lesions: 3-Year Results From the DETOUR2 Trial
Source: J Soc Cardiovasc Angiogr Interv. 2026 Jun 23;5(8):105457. doi: 10.1016/j.jscai.2026.105457 (PMC13491344; doi:10.1016/j.jscai.2026.105457)
Supplement: Supplementary file 1 [file mmc1.docx]

**Study Definitions**

**Symptomatic DVT**

Symptomatic DVT is defined as an occlusive thrombus within a deep vein with symptoms including but not limited to: pain, swelling, warmth, redness, tenderness, cramps, and/or – in extreme cases – pale/bluish decolorization of the skin of the affected extremity. Occlusive thrombus within a deep vein in the absence of any associated clinical symptoms would not constitute a symptomatic DVT. However, any venous thrombus (whether occlusive or non-occlusive) associated with pulmonary embolism will be considered symptomatic independent of presence/absence of any lower extremity complaints, as it can conservatively be assumed that the thrombus morphology may have been altered by the embolic event.

**Lost to follow-up**

Lost to follow-up was defined as failure to locate a subject after three attempts were made to contact those who do not return, followed by a final attempt via certified letter.

**MALE (Major adverse limb events)**

MALE, defined as above-ankle amputation or major additional procedures, including new bypass grafting, interposition grafting, thrombectomy, or thrombolysis.

**Primary patency**

Primary patency defined as per SVS guidelines, is as freedom from additional or secondary surgical or endovascular procedures (TLR), or the interval from the time of the original intervention until any intervention designed to maintain or re-establish patency is performed.
