# Supplementary material for: Durability of the DETOUR System for Long and Complex Femoral-Popliteal Lesions: 3-Year Results From the DETOUR2 Trial
Source: J Soc Cardiovasc Angiogr Interv. 2026 Jun 23;5(8):105457. doi: 10.1016/j.jscai.2026.105457 (PMC13491344; doi:10.1016/j.jscai.2026.105457)
Supplement: Supplementary file 2 [file mmc2.docx]

**DETOUR II Inclusion/Exclusion Criteria**

**General Inclusion Criteria**

1. Age > 18 and ≤ 90 years of age.

2. Willing and able to provide informed consent.

3. Subject is willing to undergo all follow-up assessments according to the specified schedule over 36 months.

**Clinical Inclusion Criteria**

4. Chronic, symptomatic lower limb ischemia defined as Rutherford clinical categories 3, 4, or 5.

5. Venous Clinical Severity Score < 3.

6. Subject is a suitable candidate for angiography and endovascular intervention and, if required, is eligible for standard surgical repair.

**Angiographic Inclusion Criteria**

7. Symptomatic femoropopliteal chronic total occlusions ≥ 20 cm (TASC D) that can include de novo, restenotic, or in-stent restenotic lesions;

*or*

Symptomatic femoropopliteal lesions ≥ 24 cm (total lesion length) that can include a chronic total occlusion or a ≥70% lesion that includes de novo, restenotic or in-stent restenosis (complex TASC C), by investigator visual assessment.

8. Reference vessel diameter ≥ 4.5 and ≤ 6.7 mm, by investigator visual assessment.

9. Subject has a patent popliteal artery (<50% stenosis) distal to the landing zone

10. Able to successfully access the SFA origin for entry of the crossing device.

11. At least one patent infrapopliteal vessel (<50% stenosis) with run-off to the ankle or foot.

12. A significant stenosis (≥ 50%) or occlusion of an ipsilateral, inflow artery (e.g. aortoiliac, common femoral) must be successfully treated (use of investigational treatment prohibited) prior to treatment of the target lesion. Successful treatment is defined as no complications

**General Exclusion Criteria**

1. Participating in another investigational clinical study.

2. Anticipated life expectancy less than 1 year or medical comorbid condition(s) that could limit the subject’s ability to comply with the requirements of the trial.

3. Subject has other medical, social or psychological problems that, in the opinion of the investigator, preclude them from receiving this treatment, and the procedures and evaluations pre- and post-treatment.

**Clinical Exclusion Criteria**

4. History of deep vein thrombosis on either limb.

5. Thrombophlebitis, within the previous 30 days.

6. Planned amputation of the target limb, including minor amputation.

7. Prior distal amputation (above the transmetatarsal) of the target limb.

8. Known or suspected active infection at the time of the procedure (e.g., WIfI foot infection grade 3: Severe infection. Local infection with systemic inflammatory response syndrome [SIRS]).

9. Rutherford clinical category 0, 1, 2 or 6.

10. Has acute or chronic renal disease with GFR ≤ 30 ml/min per 1.73 m2 and/or elevated serum creatinine >2.5mg/dL (220μmol/L) or on dialysis.

11. Known hypersensitivity/allergy to the investigational devices and/or required pharmacotherapy that cannot be safely managed.

12. Morbid obesity that does not allow for safe vascular access or imaging.

13. Subject has a known coagulopathy or has bleeding diatheses, thrombocytopenia with platelet count less than 100,000/microliter or INR > 1.8.

14. Requires coronary or peripheral procedure within 30 days prior to or planned within 30 days post treatment of the target lesion.

15. Has a known history of intracranial bleeding or aneurysm, myocardial infarction or stroke within the last 3 months.

16. Subject is pregnant or breast-feeding.

**Angiographic Exclusion Criteria**

17. Stent within 3 cm of SFA ostium.

18. Previous bypass surgery on the target limb.

19. Subject has significant disease or obstruction (≥50%) of the inflow tract that has not been successfully treated at the time of the index procedure (success measured as ≤30% residual stenosis, without complication)

20. Presence of aneurysm or acute thrombus in the target limb.

21. Thrombolysis of the target vessel within 72 hours prior to the index procedure, where complete resolution of the thrombus was not achieved.
