# Supplementary material for: Durability of the DETOUR System for Long and Complex Femoral-Popliteal Lesions: 3-Year Results From the DETOUR2 Trial
Source: J Soc Cardiovasc Angiogr Interv. 2026 Jun 23;5(8):105457. doi: 10.1016/j.jscai.2026.105457 (PMC13491344; doi:10.1016/j.jscai.2026.105457)
Supplement: Supplementary file 3 [file mmc3.docx]

**Subjects with DUS Occlusion**

| **Subject** | **Days to Occlusion** | **ALI Onset**  **(Yes/No)** | **Intervention** | **MALE** | **Outcome** | **Notes** |
| --- | --- | --- | --- | --- | --- | --- |
| 1 | 1161 | No | None | No | Unresolved but stable | Occlusion was found during the 36-month DUS. |
| 2 | 1106 | No | None | No | Unresolved but stable | No symptoms |
| 3 | 55 | Yes | Thrombolysis and (unknown) intervention | Yes | Resolved |  |
| 4 | 31 | No | Pharmacomechanical thrombolysis and angioplasty of the bypass graft. | Yes | Resolved | One-month DUS revealed thrombus in the bypass graft. |
| 5 | 1135 | No | Balloon angioplasty | Yes | Resolved | DUS showed occlusion of target limb vessels. |
| 6 | 1020 | No | None | No | Resolved |  |
| 7 | 34 | No | (Unknown) Interventional procedure | Yes | Unresolved but stable |  |
| 8 | 777 | No | Atherectomy and balloon angioplasty | Yes | Resolved | Subject was found to have occluded stent graft during 24- month DUS |
| 9 | 28 | No | Endovascular reintervention | Yes | Resolved |  |
| 10 | 389 | No | Only medical treatment | No | Unresolved but stable | One-year follow up DUS showed occlusion. |
| 11 | 748 | No | Thrombolysis and (unknown) intervention | Yes | Resolved |  |
| 11 | 748 | No | Thrombolysis and endovascular reintervention | Yes | Resolved | Stopped ASA and plavix. |
| 12 | 1034 | No | None | No | Resolved |  |
| 13 | 26 | Yes | Thrombolysis | Yes | Resolved | Stopped ASA and plavix. |
| 13 | 26 | Yes | Thrombolysis, thrombectomy and endovascular reintervention | Yes | Resolved | Stopped ASA and plavix. |
| 14 | 671 | No | Planned open repair bypass | Yes | Unresolved | Occlusion resulted in ischemic rest pain |
| 15 | 667 | No | None | No | Resolved | Occlusive disease is stable. Collateral blood flow is strong and provides adequate arterial flow |
| 16 | 351 | No | None | No | Resolved |  |
| 17 | 380 | No | None | No | Resolved | Occlusion found on 12 -month follow-up imaging. Graft still occluded at 36-month visit. |
| 18 | 202 | No | None | No | Resolved | Patient asymptomatic |
| 19 | 1111 | No | Thrombolysis | Yes | Resolved |  |
| 20 | 1145 | No | None | No | Resolved |  |
| 21 | 358 | No | (Unknown) Intervention | Yes | Resolved |  |
| 22 | 87 | Yes | Thrombolysis, thrombectomy and endovascular reintervention | Yes | Resolved |  |
| 23 | 41 | Yes | Thrombolysis | Yes | Resolved |  |
| 24 | 273 | No | Endovascular reintervention | Yes | Resolved |  |
| 25 | 261 | No | None | No | Resolved |  |
| 26 | 413 | Yes | Thrombolysis and endovascular reintervention | Yes | Resolved |  |
| 27 | 378 | No | Planned manual thrombectomy, thrombolytics and angioplasty | Yes | Resolved |  |
| 28 | 889 | No | None | No | Resolved |  |
| 29 | 190 | No | None | No | Resolved |  |
| 30 | 561 | No | Atherectomy | No | Resolved |  |
| 31 | 680 | No | Planned angioplasty | No | Resolved |  |
| 32 | 169 | No | Planned fem-pop bypass | Yes | Resolved |  |
| 33 | 714 | No | None | No | Resolved |  |
| 34 | 234 | No | None | No | Resolved | Found bypass graft is occluded on 6-month follow-up Duplex ultrasound that |
| 35 | 1037 | No | None | No | Resolved |  |
| 36 | 1124 | No | None | No | Unresolved but stable | Previous revasc unsuccessful and post tx angio showed occlusion |
