# Supplementary material for: Durability of the DETOUR System for Long and Complex Femoral-Popliteal Lesions: 3-Year Results From the DETOUR2 Trial
Source: J Soc Cardiovasc Angiogr Interv. 2026 Jun 23;5(8):105457. doi: 10.1016/j.jscai.2026.105457 (PMC13491344; doi:10.1016/j.jscai.2026.105457)
Supplement: Supplementary file 4 [file mmc4.docx]

**Key Endovascular and Synthetic Graft Studies**

| **Study** | Bosiers et al, 2023 | van Det et al., 2009 | Jensen et al., 2007 | Iida et al 2024 | Böhme et al 2020 | Giannopoulos et al 2021 |
| --- | --- | --- | --- | --- | --- | --- |
| **Design** | Prospective multicenter RCT | Prospective multicenter RCT | Multicenter RCT | Prospective multicenter single arm | Prospective multicenter single arm | Meta- Analysis |
| **Intervention** | Synthetic Bypass | Synthetic Bypass | Synthetic Bypass | Endovascular (Covered Stentgraft) | Endovascular | Endovascular  DES  CS  DCB |
| **Device** | Dacron or expanded PTFE | PTFE  (Gore-tex)  Dacron  (Hemashield) | Dacron | Viabahn | Viabahn | Various |
| **N** | 107 | 114 (limbs) | 208 | 321 | 71 | 3010 |
| **Mean Lesion Length** | 252.9 ± 74.9 mm | NA | NA | 23.6 ± 6.6 cm | 26.5 ± 5.31 cm | 26.9 cm |
| **12-month Patency** | 72.4% | 80.4% PTFE / 84.8% Dacron | 71% | 85.3% | 68.2% | 62% |
| **24-month Patency** | 64.0% | 64% ePTFE / 70% Dacron | 62% | 75.3% | 52.6% | 58% |
| **36-month Patency** | 57.3% | 46.4% PTFE / 55.9% Dacron | 57% | 69.8% | 40.6% | 43% |

ePTFE, expanded polytetrafluoroethylene
